# Fermentative Quality and Animal Acceptability of Ensiled Persimmon Skin with Absorbents for Practical Use in Ruminant Feed

**DOI:** 10.3390/ani10040612

**Published:** 2020-04-02

**Authors:** Shimaa Abdelazeem, Ken-ichi Takeda, Kazuhiro Kurosu, Yutaka Uyeno

**Affiliations:** 1 Graduate School of Science and Technology, Shinshu University, Minamiminowa 3994511, Japan; 17st502e@shinshu-u.ac.jp (S.A.); ktakeda@shinshu-u.ac.jp (K.T.); 2 Faculty of Veterinary Medicine, South Valley University, Qena 83523, Egypt; 3 Nippon Paper Industries Co., Ltd., Tokyo 1140002, Japan

**Keywords:** absorbents, fruit byproducts, *Lactobacillus buchneri*, methane, palatability

## Abstract

**Simple Summary:**

Although persimmon skin (PS) has a high nutrition content, it loses much of its nutrient value through effluent, which hampers its use as a feed. To reduce effluent production during ensiling, we tested three kinds of moisture absorbents (kraft pulp, wheat bran, and beet pulp). We evaluated five types of table scale silages: persimmon skin only, persimmon skin plus *Lactobacillus buchneri* inoculum, and persimmon skin plus *L. buchneri* plus each absorbent in turn. We observed low effluent and gas loss in absorbent-treated groups, implying a positive contribution of these absorbents to fermentation quality. Further, we performed an in vitro culture test of PS silage supplemented with an absorbent mixture. The proportion of methane to total gas decreased in the ensiled group compared with that in the other groups. Finally, we conducted a preliminary feeding experiment on dry ewes to assess the palatability of PS with a partial substitution of up to 20% dry matter, and found no adverse effect on feed intake and acceptability. Our results highlight the potential use of PS as a part of ruminant feed.

**Abstract:**

Persimmon skin (PS), while representing an attractive feed source, requires an appropriate preservation procedure to increase its shelf life. We assessed the fermentation quality, in vitro ruminal incubation, and intake of persimmon skin silage ensiled with different dry absorbents. We prepared the silage on a table scale (Experiment 1) and evaluated five different mixtures: PS without an additive, PS plus *Lactobacillus buchneri* inoculum (LB), and PS plus LB plus each of the absorbents kraft pulp, wheat bran, or beet pulp. We opened the laboratory bags, kept at 25 °C, at 0, 14, 28, and 60 days for fermentation quality and chemical analysis (*n* = 3 for each measurement). Further, with an in vitro rumen simulated cultivation study (Experiment 2), we evaluated the fermentation pattern of PS with a mixture of two absorbents (kraft pulp and wheat bran) either raw (no fermentation) or ensiled (*n* = 4 for each treatment). Finally, we conducted an in vivo experiment using six dry ewes assigned based on their body weight to two experimental groups in a crossover design of two periods (Experiment 3). We fed a control group a 100% basal diet (tall fescue hay and concentrate mixture) and ensiled PS (PSS) group, a 20% dry matter substitution of tall fescue with PS silage mixed with kraft pulp as the sole absorbent. The results of Experiment 1 show, regardless of the absorbents used, the effluent volume of the lab bags was lower in absorbent-treated groups (*p* < 0.001). In Experiment 2, the condition of the PS with absorbents (raw or ensiled) did not affect the total gas production (*p* > 0.05), but we observed an increased propionate proportion in PSS with absorbents compared to basal diet (*p* = 0.019). The proportion of methane to the total gas in PSS group was considerably reduced compared with that in the other groups (*p* < 0.001). As we did this incubation study with a single run, a more detailed evaluation in the future would verify these observations. In the animal trial (Experiment 3), dry matter intake was similar between groups (*p* > 0.05), but ewes spent a shorter time eating in the PSS-fed group (*p* = 0.011). Here we present the practical use of PSS as part of ruminant feed in which dry absorbents prevented dry matter loss.

## 1. Introduction

Persimmon (*Diospyros kaki*), a widely distributed fruit in Asian countries, is prevalent in the food industry in Japan and South Korea, accounting for more than 200 kilotons of production per year [1]. It is sometimes consumed in a dried form, as Hoshigaki, and the preparation process is accompanied by up to 20% of persimmon skin (PS) production after peeling the skin of persimmon. Persimmon skin is high in nutritional value because it is rich in soluble carbohydrates and polysaccharides, such as pectin, which represent a suitable energy source in animal feed. However, the high moisture content and seasonal production of PS are obstacles for its subsequent use. In silage preparation, lactic acid bacteria (LAB) have been used to maintain the silage quality, since a high concentration of lactic acid and acetic acid is known to prevent the growth of some detrimental microorganisms (for instance, yeast and fungi), which can lead to silage deterioration [2,3]. After testing LAB strains, one strain (*Lactobacillus buchneri*) was identified to lead to the production of high-quality PS silage (PSS) [4,5]. Another important issue in silage preparation, especially when ensiling byproducts with high moisture content, is to minimize effluent loss. In this sense, a variety of feedstuff and nonfeed materials—including beet pulp (BP), wheat bran (WB), wheat straw, newspaper, and paper waste—have been investigated as dry absorbents [6,7,8]. Because of their high fiber content and water retention capacity, most of these dry absorbents are, to variable degrees, effective in preventing silage effluent. We hypothesized that the inclusion of dry absorbents is an effective means of reducing the effluent problem in PSS. In particular, kraft pulp (KP), made from wood chips through a cooking process that selectively removes lignin, has been evaluated as a replaceable feed material for other more readily digestible carbohydrate sources [9,10]. Notably, was the use of KP as an absorbent for PS silage, which would help optimize carbohydrate balance and therefore has been evaluated as a particularly suitable initiative for solid feed for calves [11].

We observed in a previous paper [4] that the inclusion of ensiled PS (without absorbent, at that time) decreased in vitro methane generation and that the change was likely dependent on the carbohydrate profile and on the compositional changes in the ensiled byproduct. From this viewpoint, we conceived that compositional changes in absorbents occuring during ensiling may affect in vitro rumen fermentation profile and methane generation. Furthermore, in vitro assessment does not consider some practical issues, such as palatability. In particular, PS contains a considerable proportion of certain bioactive compounds such as condensed tannin [1], which produces an astringent sensation in the mouth [12]. Therefore, for its practical use as a feedstuff it is necessary to assess that no effect on palatability or intake occurs.

The presented study aimed to proceed the applicability of PS for practical feed use, intending to solve concerns and questions described above. We first intended to conduct a table scale ensiling experiment that covered our idea and to what extent it is beneficial using absorbent into PS silage preparation (Experiment 1). We subsequently conducted an in vitro rumen cultivation test to determine the effects of PS with absorbent on fermentation parameters when it was ensiled or not (Experiment 2). Furthermore, to date, no published work for practical feeding test applying PS has been conducted. Additionally, we performed a palatability evaluation for studying the effect of partial substitution (20%) of dry matter (DM) content by PSS, which was assessed by feed intake and feeding behavior (Experiment 3).

## 2. Materials and Methods

### 2.1. Experiment 1. PS Silage

#### 2.1.1. Silage Preparation

We obtained PS from a local processing provenance in Nagano prefecture, Japan. In this study, we used three types of commercially available dry absorbents: kraft pulp (KP, Nippon Paper Industries Co., Ltd., Tokyo, Japan), wheat bran (WB, Nippon Flour Mills Co., Ltd., Tokyo, Japan), and beet pulp (BP, Nippon Beet Sugar Manufacturing Co., Ltd., Tokyo, Japan). We used *L*. *buchneri* NBRC107764 inoculum (NITE Biological Resource Center, Tokyo, Japan). The experimental treatments were as follows: PS without additive (CON), PS plus *L. buchneri* inoculum (1.0 × 10^9^ colony forming units (CFU)/kg PS, LB) and LB plus absorbents: 416 g KP/kg PS (DM basis; KP), 788 g WB/kg PS (WB), or 402 g BP/kg PS (BP). The inclusion rate of each absorbent depended on its water retention capacity, based either on previous literature values (WB and BP) [7] or on information provided by the manufacturer (KP).

The procedures for bag silage preparation and fermentation control were described in our previous reports [4,5]. Briefly, we cut PS to a short length (5–10 cm) before combining the inoculum and absorbents and mixed all materials well to ensure even distribution. We packed approximately 20 g of each treatment in polyethylene bags (15 × 22 cm), which we tightly heat-sealed under vacuum (SQ-205S; Asahikasei Packs Co. Ltd., Tokyo, Japan) and then incubated at 25 °C. Fifteen bags per group were prepared for this ensiling experiment.

#### 2.1.2. Chemical and Microbial Analysis of Silage

On the day of ensiling (day 0), we took two bags per treatment for chemical and microbiological analysis. Thereafter, we evaluated the fermentation profile by opening three bags for each group on day 14, day 28, and day 60 of the ensiling process. To detect the fermentation endproducts, we prepared water extract from silage by adding 180 mL distilled water into bags and homogenizing for 1 min, thereafter storing it for 2 h at 5 °C. We used a portion of the water extract to measure the pH using a pH meter (Model D-51, Horiba Co. Ltd., Kyoto, Japan). We divided another portion from the silage extract into two subsamples. One subsample was centrifuged at 10,000 × g for 10 min, and the collected supernatant was used for the analysis of organic acids by high-performance liquid chromatography according to the same protocol described in Section 2.2 and for ammonia nitrogen determination, using a commercial kit (F-Kit Ammonia, Roche Diagnostics, Tokyo). We used the second subsample (uncentrifuged) for microbial counts after serial dilution using a phosphate buffer solution, using the diluted sample to determine LAB counts (using MRS agar [Oxoid, Basingstoke, UK] incubated at 30 °C for 3 days) and yeast counts (using chloramphenicol-added potato dextrose agar, incubated at 25 °C for 7 days).

Another three silage bags were weighed and opened on day 60. After opening the bags, gas loss was calculated, as described in Section 2.4, and then effluent output was measured using a graduated cylinder. Remaining contents in the bag were used for determination of dry matter (DM) and neutral detergent fiber (NDF). Dry matter content was determined by oven drying at 100 °C for 3 h, as was the condition for DM determination of silage effluent. Methods for proximate analyses (crude ash [942.05], crude fat [945.16], and crude protein [976.05]) were followed to standards described by AOAC [13]. We analyzed neutral detergent fiber (NDF) following the method described previously [14] using heat-stable alpha-amylase without the use of sodium sulfite, and presented the NDF result, including the ash. Chemical analyses (DM, NDF, and fermentation endproducts) were conducted twice for each bag (and its water extract), and the mean value of these two measurements per bag was used for the statistical treatment. For reference information, we calculated soluble protein and fiber compartments of test material mixtures prior to silage fermentation with reference to either proximate analysis data (PS and KP) or a standard table of feed composition (BP and WB) [15] (Supplementary Table S1).

### 2.2. Experiment 2. Batch Culture Trial

Judging from results of the ensiling experiment, we determined to apply a mixture of KP and WB as the absorbent mixture (ratio approximately 1:1; reason of the mixture was simply due to unavailability of KP in a sufficient amount) for laboratory-scale PSS preparation. We mixed 9.2 kg DM of PS (30 kg as fresh matter [FM] weight) with a 400 g absorbent mixture /kg PS [DM basis]. Before ensiling a sample of 100 g (FM basis) was taken and air dried for further in vitro incubation tests. Then, the mixture was ensiled; for this it was inoculated with 1.0 × 10^9^ CFU/kg PS *L. buchneri*, transferred to a plastic bag (size 45 L), followed by foot treading, and then tightly sealed it. We ensiled the mix in a temperature-controlled room set at 25 °C for 60 days (November to January; fermentation properties are available in Supplementary Table S2). We air dried part of this PSS sample at 60 °C for 16 h, ground them, and kept them at −20 °C until using them in the in vitro culture test. The remainder of the lab scale silage was subject to feed animals in the preliminary feeding test (pretest) described in the next section.

We conducted an in vitro incubation experiment using unfermented and ensiled PS plus absorbents, basically following those described in our previous report [4]. We performed this test in a single run using four replicate bottles. We set experimental substrates as (1) the basal feed group, based mainly on forage material (commercial timothy hay; DM, 850 g/kg FM; crude protein [CP], 110 g/kg DM; digestible energy [DE], 12.5 MJ/kg DM; calculated value) and concentrate (commercial concentrate; DM, 870 g/kg FM; CP, 220 g/kg DM; DE, 15.5 MJ/kg DM; calculated value) at ratio 80:20 (DM basis); (2) the PS and absorbent group (PSA) containing basal feed with air dried, unfermented PS with the absorbent (at ratio 82:18 [DM basis] and 67:33 [fresh basis]; adjusted to level in our previous paper [4]); and (3) the ensiled PS group (PSS), including basal feed with ensiled persimmon skin with the absorbent (at same ratio as PSA). The compositions of PSA and PSS were as follows: DM, 383 g/kg FM; CP, 36 g/kg DM; NDF, 350 g/kg DM for PSA, and DM, 370 g/kg FM; CP, 39 g/kg DM; and NDF, 440 g/kg DM for PSS, respectively.

Rumen fluid samples for the cultivation test were collected from three beef steers (Japanese Black) fed concentrate (5.0 kg DM/day) and a 1.4 kg DM mixture of forages comprising Italian ryegrass (*Lolium multiflorum*) and orchard grass (*Dactylis glomerata*) via a rumen fistula immediately before the morning feeding, under approval obtained from the Committee for Animal Experiment, Shinshu University (R105001). The collected fluids were equally mixed and filtered through four layers of cheesecloth, diluted with artificial saliva (McDougall buffer) at a 2:1 (buffer:the rumen fluid) ratio, and used within 2 h of collection. We transferred a 40 mL aliquot of the diluted rumen fluid into a 100 mL serum bottle containing experimental substrate (1.0 g) flushed continuously with CO_2_ gas. We sealed the bottles with a rubber plug and aluminum cap after flushing the headspace with CO_2_ gas, and then incubated them at 39 °C for 24 h, with periodical removal of headspace gas by a needle attached cylinder.

After incubation, we measured the total headspace gas production, methane proportion, volatile fatty acid (VFA) content, and ammonia nitrogen. Headspace gas production was determined by cumulating periodical gas removal. Proportion of methane was analyzed by a gas chromatography system (Shimadzu GC-8A) following previous reports [16], using a steel column (2 m, 3 mm, Shincarbon-ST; Shinwa Chemical Industries Ltd., Kyoto, Japan) and a thermal conductivity detector (TCD, 210 °C). Argon gas was used as the carrier gas at a 50 mL /min flow rate. A 0.5-mL aliquot of headspace gas was injected by a gas-tight syringe. Methane gas volume in milliliters was calculated using a standard gas mixture, calculated using an attached software (ChromatoPak). We analyzed VFA content by high-performance liquid chromatography, using an LC-2000 system (JASCO Corporation) under the conditions of those given in our previous report [11], under the following conditions: column, Inertsil ODS-3 250 mm × 4.6 mm (GL Science Co. Ltd., Tokyo, Japan); oven, 40 °C, mobile phase, 10% acetonitrile−0.02% perchloric acid; flow rate, 1 mL/min; and detection, 210 nm absorbance. Operation control, peak detection, and quantification were performed using a software attached to the system (ChromNAVI). Ammonia nitrogen was determined by a commercial kit (F-kit, Roche Diagnostics, Basel, Switzerland) according to the instruction.

We also quantified the total bacterial count, the archaea, and the *Fibrobacter* from the batch culture test using a real-time polymerase chain reaction method. We performed DNA extraction for microbial analysis using the QIAamp DNA Stool Mini Kit (Qiagen, Hilden, Germany), following the manufacturer’s recommendations and stored the obtained DNA at −20 °C until analysis. For the real-time PCR, we used the primer sets Eub338F (ACTCCTACGGGAGGCAG) and Eub522R (ACGTCRTCCMCNCCTTCCTC) for total bacteria count, qmcrA-F (TTCGGTGGATCDCARAGRGC) and qmcrA-R (GBARGTCGWAWCCGTAGAATCC) for prokaryotic archaea count, and Fs1f (GTTCGGAATTACTGGGCGTAAA) and Fs1r (CGCCTGCCCCTGAACTATC) for *Fibrobacter* count using CFX96™ Real-Time system (Bio-Rad Inc., Hercules, CA, USA) and a SYBR(R) Premix Ex Taq™ Kit (Takara Bio Inc., Otsu, Japan) for the real-time PCR. We performed cycling conditions with 40 cycles; each cycle included denaturation at 95 °C for 10 s, annealing at 60 °C for 20 s, and extension at 72 °C for 30 s, followed by dissociation curve analysis to confirm that we had obtained the expected PCR endproducts.

### 2.3. Experiment 3. Feeding Trials

#### 2.3.1. Pretest

We conducted two feeding experiments to assess its acceptability in partial substitution with PSS. Because there was no comparable data on the provision of PSS (or even PS) on ruminants, we firstly conducted a preliminary experiment (referred to as pretest) on dry ewes. We conducted all animal management and handling following the guidelines of Shinshu University, with the approval for animal experiment that was the same as above. In this test, we assigned six multiparous/primiparous ewes (Suffolk breed) with an average body weight (BW) of 69 ± 8 kg (mean ± SD) in a 3 × 3 design of two animals each and fed each group one of the following diets (Supplementary Table S3; 11 days for the adaptation and 3 days for the collection of data regarding feed intake): (1) control diet without PSS; or a diet with (2) 12% or (3) 25% DM replacement by the PSS product that was used in the in vitro rumen cultivation test described above. In the test, we observed no feed refusal during the entire experiment when PSS was offered (up to 25% DM), and daily DM intake did not differ (*p* = 0.582, one-way ANOVA) among the groups (1.27 ± 0.06 kg/day, mean ± SE).

#### 2.3.2. Main test

Based on the observation from pretest, we decided to mix PSS with the absorbent as 20% DM replacement to evaluate the palatability of the mix and animal behavior toward PSS in the main teat. We collected PS in November 2018 from the production area in the Nagano Prefecture, Japan. We used KP as dry absorbent and inoculated silage with *L. buchneri* (1.0 × 10^9^ CFU/kg the ensiled mixture). We mixed the freshly collected PS (30 kg as fresh matter [FM] weight) with 400 g KP/kg DM PS, and packed the mixed material in large doubled polyethylene bags (size 45 L), followed by foot treading, and then tightly sealed it to be used in the feeding trial. We monitored the fermentation profile (silage pH, LAB, and yeast count) once per week as described in previous section, and values at the endpoint (three weeks) were summarized in Supplementary Table S2.

We assigned six dry ewes (Suffolk breed) based on BW, with initial BW of 79 ± 7 kg (mean ± SD) to two groups in a crossover design. We kept each animal individually in a cage fitted with isolated feed buckets and a water container and supplied every cage with a mineral block (per 100 g: 39.9 g Na, 1.33 mg Fe, 106 mg Ca, 102 mg Mg, 0.04 mg Mn, and 56.4 mg K). We fed each group a ration with either 0% or 20% PSS in two consecutive experimental periods with three animals per treatment (amounting to six animals per treatment). To supply the maintenance requirements for sheep according to the Japanese guidelines, we formulated the two diets to have practically the same level of energy and CP (Table 1), by adjusting proportions of the three feed materials (PSS, tall fescue hay, and concentrate mainly consisted of maize, soybean meal, and barley) [17]. Before offering it to the animals, we chopped tall fescue hay with a mechanical chopper to 8 cm. We distributed the diets twice per day at 0800 h and 1600 h, collecting and weighing feed refusals every day. Each period extended for 14 days and included 10 days for adaptation, followed by 4 days for measurement and collection. During the adaptation period, we gradually increased the amount of PSS to 6% (1st and 2nd day) and 13% (3rd and 4th day) to substitute tall fescue hay, up to 20% on a DM basis (after 5th day). Interval for washout was set for three days between diet switching.

For behavior measurement, we recorded time spent eating by direct (i.e., without camera usage) monitoring for two hours after each feed offering timing. For other timing periods, we applied two sets of portable time-lapse cameras with Illumi-Night Sensors to facilitate video capturing during the nighttime; each one recorded the behavior of three animals. Cameras were set on motion detector mode for three days to capture their behavior in each experimental period, to define time spent eating, and the onset of a rumination bout as the time when regurgitation occurred—namely, when a bolus came up the esophagus and reached the mouth. The end of a rumination bout was the minute the last bolus was swallowed.

### 2.4. Calculations and Statistical Analysis

In Experiment 1, gas loss of silage bags after 60 d of ensiling was calculated as follows:

(1) GL (g)=BWE (g)−BWO (g)

where GL is the gas loss (g), BWE is the bag weight at the ensiling (g), and BWO is the bag weight after the opening (g).

Analysis of variance (ANOVA) was applied for pH, yeast, lactic acid bacteria, DM, and NDF in the ensiling experiment. The following model was used:

(2) $$Y_{\mathrm{ij}}=µ+\alpha_{i}+\beta_{j}+{\left(\alpha \beta \right)}_{\mathrm{ij}}+e_{\mathrm{ij}}$$

where Y_ijk_ = observations for dependent variables; µ = overall mean; α_i_ = the fixed effect of treatment; β_j_ = the fixed effect of time; (αβ)_ij_ = the interaction between treatment and time; and e_ij_ = the residual error. Numbers of j were different among items (four for pH, yeast, and lactic acid bacteria; two for DM and NDF). We analyzed data of the in vitro cultivation and of the fermentation characteristics and chemical composition at the endpoint (60 days) except for those described above using a one-way ANOVA and Tukey’s test to detect the presence of significant differences between treatment means. All results from the in vivo trial (main test of Experiment 3) were stated as the mean ± SE. We applied Wilcoxon signed-rank tests for comparisons between order effects and period effects but found no significant difference. Differences between means were analyzed with independent samples t-test. All statistical procedures were conducted using Statistical Packages for the Social Sciences (SPSS). A *p*-value of less than 0.05 was considered statistically significant.

## 3. Results

### 3.1. Experiment 1. PS Silage

As shown in Table 2, BP-treated silage had a lower pH than that in other groups at the beginning of the ensiling process. After 14 days of ensiling, the pH dropped dramatically to less than 4.0 for all treatment groups. After 60 days of ensiling, we detected a higher pH in the WB-treated group compared to that of the other groups (*p* < 0.001). While we determined a certain volume of lactobacilli in control, *L. buchneri* inoculation resulted in an increased LAB count relative to the control group (*p* < 0.001). Moreover, in WB and BP, the yeast count was under the detection level (×10^2^ cfu/g) from 14 days. After 60 days of ensiling, we detected no yeast growth in any LB-treated groups, with or without absorbents.

We detected only lactate and acetate but noted neither propionate nor butyrate in any of the silages (Table 3). Lactate levels were significantly higher in WB and BP than in other groups. Acetate levels in the LB-inoculated groups (with or without the absorbents) were marginally higher than those in the control group. We detected no significant difference in terms of ammonia nitrogen concentration between the groups.

Dry matter and NDF of each group before and at 60 days of silage fermentation are shown in Table 3. Regarding other nutrients, initial content of crude protein (g/100 g DM) was 3.6 ± 0.1, 3.7 ± 0.2, 3.2 ± 0.2, 6.4 ± 0.3, and 3.4 ± 0.2 for CON, LB, KP, WB, and BP, respectively, but we did not trace crude protein content after fermentation. Initially, the inclusion of KP, WB, and BP increased the DM content compared with the control and LB groups. The NDF proportion seemed to increase in the three absorbent groups (KP, WB, and BP) at the beginning (*p* < 0.001). The NDF proportion per DM decreased in the CON, LB, and BP groups compared with that pre-ensiling, whereas it increased during ensiling in the KP group. The absorbent-treated silages (KP, WB, and BP) produced a negligible amount of effluent, whereas the other groups (CON and LB) generated up to 21.3 mL (CON) and 17.5 mL (LB) of effluent per 100 g FM, respectively, accounting for 12 % DM loss in the effluent. The control group, compared with two of the absorbent-treated groups (KP and BP), had the highest gas production during ensiling (*p* = 0.045).

### 3.2. Experiment 2. Batch Culture Trial

No significant difference was determined in the total gas production (*p* = 0.118) and in the total VFA production (*p* = 0.108) among groups (Table 4). Propionate proportion in total VFA significantly increased in PSA and PSS groups compared to that in the control (*p* = 0.019), and a significant decrease in acetate proportion in PSA group (*p* < 0.001) compared to control and PSS was observed. In the PSA group, we noted a significant decrease in ammonia in comparison with that of the control concentration (*p* = 0.004). In PSS, the proportion of methane to total gas was considerably reduced in relation to that in the other groups (*p* < 0.001). Compared with the fresh material, PSS showed a lower proportion of *Fibrobacter succinogenes* (*p* < 0.001) and tended a low proportion of archaeal 16S rRNA in the total community.

### 3.3. Experiment 3. Feeding Trials

Persimmon skin silage employed in Experiment 3 was comparable in quality to those of bags of KP group in Experiment 1, as well as a laboratory scale preparation for the preliminary feeding test (Supplementary Table S2). In addition, we detected no effluent leakage in the silage package.

During this short-term experiment, the dietary treatment had no significant effect on BW change. PSS inclusion in sheep feed did not affect DM, CP, and NDF intake (Table 5). The result indicated that the PSS-fed group spent less time in eating per DM ingestion (*p* = 0.011), but no significant difference in rumination time per DM ingestion.

## 4. Discussion

### 4.1. Experiment 1. PS Silage

Preventing effluent output comprises two approaches: increasing DM at pre-ensiling and absorbing water leaked during ensiling. The former idea has been examined in preceding studies, for instance, Fransen et al. evaluated absorbents, such as rolled barley, beet pulp, or crushed alfalfa cubes to nonwilted grass forage silage, and found a negative, quadratic relationship between pre-ensiled DM and grass silage effluent [6]. Another group reported that the addition of 9% of bran to vegetable waste (12% DM) decreased effluent output [18]. Maintaining silage at high DM also contributes to preventing toxigenic fungi and mycotoxins [19].

Taking results obtained in a previous study into account [4], we have applied one silage inoculant, *L. buchneri* culture. *Lactobacillus buchneri* is known as having the unique ability of anaerobically converting lactic acid to acetic acid and 1,2-propanediol—both of which are known as antifungal agents [20]; thus, it is regarded as an effective silage inoculant that prevents aerobic deterioration [21,22,23]. In the present study, LAB inoculation may have affected silage fermentation pattern in either lactate, acetate, or both, compared with the result in CON. Further, two other metabolites, 1-propanol and propionate acid, are sometimes found in *L. buchneri* treated silages [24]. According to previous reports [25], these compounds are generated from 1,2-propanediol by *Lactobacillus diolivorans*, which is taxonomically close to *L. buchneri*. While we could not detect propionate in PSS, it is worth analyzing detailed lactobacilli population in species level, in order to conceive what is a probable mechanism to prevent silage deterioration. In contrast, gaseous loss was particularly high in the absorbent-free groups, a result partly due to low DM (i.e., high moisture content), which promoted unnecessary fermentation by other bacteria, yeast, and fungi [7]. As PS includes a high proportion of readily fermentable sugars [5], to implement another option in addition to LAB would be desirable for preventing the silage from microbial deterioration. In this regard, supplying absorbent to PS was an effective means of increasing DM. The addition of dry absorbents, because of water retention capacity, successfully reduced effluent volume to zero and a prevention of DM loss in the effluent that accounted for around 12 g/100 g DM, which presumably involved soluble sugar and protein, and some insoluble nutrients described below. Notably, we cut this material into around 5–10 cm in the bag test, retaining a larger surface area per material weight compared to that of practically generated PS having a length of 50–80 cm. This difference would have enhanced effluent output in the CON group.

Notably, both the treatment (absorbent inclusion) and ensiling process affected the chemical composition of the ensiled material. Although not significant, a relationship between treatments and analysis timings (pre-ensiling and at day 60) in the NDF was remarkable. We observed that at day 60, NDF decreased in CON and LB groups, possibly because of hemicellulose breakdown in PS portion (accounted for 141 g/kg DM [See Supplementary Table S1]) caused by hydrolysis resulting from fermentation acid. Part of such hemicellulose-derived small molecules presumably dripped off with leaked water. In case of the BP group, an NDF decrease was also remarkable, possibly due to degradation of hemicellulose compartment in BP. On the other hand, as KP comprises almost 100% cellulose [9] and contributed to increase in cellulose proportion (up to 185 g/kg DM [See Supplementary Table S1]), which could escape intensive degradation during ensiling, the relative proportion of NDF in total DM was supposed to increase after fermentation.

The ammonia nitrogen level was also low regardless of inoculant and absorbent inclusion, a result in agreement with that of a previous study [26] reporting that barley silage treated with fermented persimmon extract had low ammonia nitrogen concentration. It was likely because of relatively little proportion of crude protein in PS (around 30 g/kg DM). On the other hand, it may also be due in part to the action of tannins in PS, which have a high affinity to bind protein and protect it from proteolytic microorganisms [27].

Overall, all three kinds of absorbents contributed to an improvement in ensiling quality in PS with respect to lowering both gas production and effluent. Judging from results of the ensiling experiment, we assumed that the three kinds of absorbents tested possessed essentially similar properties from a practical point of view. Accordingly, in larger scale silo preparations we used the inoculum and KP as a main composite of the absorbent to make the silage balanced in view of carbohydrate proportion (fiber and readily fermentable sugar).

### 4.2. Experiment 2. Batch Culture Trial

We subsequently aimed to reveal the change in in vitro rumen culture when PS with absorbent was mixed to a forage-based feed ration and to elucidate the effect of the ensiling of the PS-absorbent on the cultivation. Interestingly, some changes in the incubation parameters were observed between groups of diets with ensiled- and raw-PS-absorbents (Table 4). The PSA contained raw PS plus absorbents (KP and WB in this case), all of which were not yet degraded by ensiling. In a previous study, we observed that addition of raw PS into diet affected in vitro rumen incubation product decreasing acetate and increasing propionate and butyrate proportions [4]. These results were basically in accordance with results in PSA group of the present experiment (i.e., PS plus KP), although we did not observe the proportional increase in butyrate. A significant decrease in ammonia in the PSA group was better attributed to effective utilization of nitrogen source by bacteria using energy source (fermentable carbohydrates) remaining in the fresh material, rather than the effect of tannin [12].

Decrease in in vitro methane generation brought by the PSS group was maintained, as it was reported previously as well, even when absorbents were added. It was in good accordance with a tendency of low proportion of archaeal 16S rRNA in the total community. We previously conceived that partially-degraded fiber generated from ensiling may provide more suitable fermentation substrates for some bacteria involved in fiber digestion, introducing changes in the fermentation product to limited hydrogen generation, which was expected to reduce methane production [4]. Additionally, in an in vivo trial [11], feeding KP to calf resulted in a significant increase in *Fibrobacter* proportion; a similar result was obtained in the PSA group. However, the *Fibrobacter* proportion decreased in PSS, which likely had relatively higher NDF content per DM than the other two groups. As recently shown in in vivo and in vitro digestibility evaluation trials of fermented feeds containing food byproducts [28,29,30], the ensiling process improved fiber digestibility; thus, a similar change was expected to occur in PSS compared with PSA (before ensiling matter). Taken together, although we did not determine detailed fiber profile (acid detergent fiber and acid detergent lignin), it was supposed that during ensiling, the absorbent material changed proportion in structural and nonstructural carbohydrates particularly in the case of KP, thereby affecting the rumen microbial profile. When the *Fibrobacter* population was low after incubation, fiber degradation could mainly be participated by other kinds of fiber-degrading bacteria; for instance, *Ruminococcus* species. Because *Ruminococcus* is known to release H_2_ as a metabolite of cellulose breakdown [31], the hydrogen may have reduced CO_2_ to methane, which is however, inconsistent with present results of decreasing methane generation in PSS. Another interpretation of this result would be that other types of rumen bacteria were activated and collaborated together to degrade various nutrients, including fiber, as it usually occurs in the actual rumen community [32]. Therefore, the ensiling of PS plus absorbent might offer favorable conditions for the growth of non-fiber-degrading bacteria that preceded fiber-degrading bacteria and it would invoke more rigorous fermentation.

The drawback of the present in vitro incubation experiment was also that we conducted only one run to obtain results, whereas at least three independent incubation runs are recommended [33]. With inclusion of the matter of multiple incuvation runs, evidence for the community dynamics provided by PSS with absorbents has yet to be produced by detailed analysis of nutrients breakdown during incubation (i.e., digestibility), and overviewing changes in microbial community.

### 4.3. Experiment 3. Feeding Trials

In addition, we aimed to perform a palatability evaluation for studying the effect of partial substitution (20%) of DM content by PSS. To the best of our knowledge, there was only one animal feeding study conducted by Kim et al. [34], who demonstrated that when feeding finishing pigs diets containing up to 7% fermented persimmon shell, which positively affected measurements regarding both growth performance and meat quality. In the present study however, we did not detect remarkable changes in the body weight of animals in either groups since adult ewes were applied. Sheep spent a significantly shorter time eating in the PSS-fed period than the control period, as we often observed that they ate the PSS feed very quickly. There are some probable reasons of good palatability for animals; a high proportion of readily fermentable carbohydrates (as partly explained rich proportion of NFC shown in Table 1) may have remained in PSS, although it was expensed during ensiling at a certain amount. Another reason of the palatability would be simply higher moisture content in PSS ration, which helped smooth intake by animals. These factors may have exceeded negative factors within PS such as astringency. No significant change in the rumination time per DM intake was likely due to the same level of NDF intake between the groups, partly attributed to fortifying fiber from the absorbent. From this viewpoint, including fibrous materials such as KP may be a favorable option to alleviate decreases in fiber contents of feed that include a certain amount of PS. Further feeding tests are in progress to elucidate the nutritional properties and ruminal fermentation kinetics occurring in response to PSS supplementation in ewe’s feed.

## 5. Conclusions

Using various dry absorbents for moisture adjustment of PSS, we decreased the effluent loss to a negligible volume. Sheep consumed a ration partially substituted with PSS (up to 20%) without adverse effect on feed intake and palatability. Here, we demonstrated the potential use of PS as part of the ruminant feed. Detailed animal feeding trials as well as intensive in vitro incubation experiments are required to determine whether feeding PS affects feed efficiency or how it impacts enteric methane emission.

## Figures and Tables

**Table 1:** The chemical composition of the main dietary ingredients and tested feed in the feeding trial (main test, Experiment 3).

Items	PSS	Tall Fescue Hay	Concentrate Mixture	Test Feed	
				Control Period	PSS Period
DM (g/kg FM)	320	890	870	884	668
OM (g/kg DM)	945	935	960	941	943
CP (g/kg DM)	30	45	220	90	85
CFat (g/kg DM)	16	10	25	14	15
NDF (g/kg DM)	533	650	100	540	517
NFC (g/kg DM)	366	230	615	297	326
TDN (g/kg DM)	580	500	840	580	590
DE (MJ/kg DM)	10.9	9.2	15.6	10.8	11.0

CFat, crude fat; CP, crude protein; DE, digestible energy; DM, dry matter; NDF, neutral detergent fiber; NFC, nonfiber carbohydrates (calculated by OM - [sum of CP, CFat, and NDF]); OM, organic matter; PSS, persimmon skin silage; and TDN, total digestible nutrients. DE and TDN were calculated in accordance with a feeding standard [15]. For the concentrate mixture, we used a commercial concentrate for dairy heifers consisted mainly of corn, barley, soybean meal, rapeseed oil meal, wheat bran, calcium bicarbonate, and vitamins. Test food was an example ration for 80 kg BW ewes; compositional values were calculation results based on those in each material.

**Table 2:** Time course of fermentation characteristics (pH, yeast, and lactic acid bacteria) of persimmon skin (PS) supplemented with absorbents during ensiling (Experiment 1).

Ensiling Period	Treatment					Contrast
	CON	LB	KP	WB	BP	
pH						
Day 0	6.32 ± 0.07 ^aA^	6.27 ± 0.02 ^aA^	6.37 ± 0.04 ^aA^	6.31 ± 0.01 ^aA^	5.79 ± 0.03 ^bA^	Trt, *p* < 0.001
Day 14	3.72 ± 0.02 ^aB^	3.63 ± 0.02 ^aB^	3.68 ± 0.02 ^aB^	3.50 ± 0.03 ^bC^	3.71 ± 0.01 ^aBC^	Time, *p* < 0.001
Day 28	3.63 ± 0.04 ^bcB^	3.57 ± 0.01 ^cBC^	3.68 ± 0.03 ^bB^	3.58 ± 0.01 ^cC^	3.76 ± 0.02 ^aB^	Trt×Time,
Day 60	3.58 ± 0.04 ^cB^	3.53 ± 0.02 ^cC^	3.56 ± 0.01 ^cC^	3.78 ± 0.01 ^aB^	3.68 ± 0.01 ^bC^	*p* < 0.001
Yeast (log10 CFU/g FM)						
Day 0	4.53 ± 0.05 ^B^	4.38 ± 0.01 ^A^	4.25 ± 0.05 ^A^	4.43 ± 0.02 ^A^	4.23 ± 0.07 ^A^	Trt, *p* < 0.001
Day 14	5.35 ± 0.40 ^aA^	3.18 ± 0.18 ^bB^	2.05 ± 0.05 ^cC^	ND ^dB^	ND ^dB^	Time, *p* < 0.001
Day 28	5.00 ± 0.70 ^aAB^	3.38 ± 0.02 ^bB^	2.51 ± 0.03 ^bB^	ND ^cB^	ND ^cB^	Trt×Time,
Day 60	4.25 ± 0.23 ^aBC^	ND ^bC^	ND ^bD^	ND ^bB^	ND ^bB^	*p* < 0.001
Lactic acid bacteria (log10 CFU/g FM)						
Day 0	6.14 ± 0.12 ^cC^	7.27 ± 0.03 ^aC^	7.35 ± 0.04 ^aC^	6.93 ± 0.00 ^bC^	7.32 ± 0.03 ^aC^	Trt, *p* < 0.001
Day 14	7.89 ± 0.02 ^cA^	8.52 ± 0.09 ^aAB^	8.95 ± 0.03 ^aA^	8.37 ± 0.04 ^bA^	8.09 ± 0.07 ^bAB^	Time, *p* < 0.001
Day 28	7.77 ± 0.07 ^cA^	8.86 ± 0.02 ^aA^	8.92 ± 0.05 ^aA^	8.04 ± 0.07 ^bB^	8.17 ± 0.06 ^bA^	Trt×Time,
Day 60	6.48 ± 0.17 ^cB^	8.45 ± 0.01 ^aB^	8.50 ± 0.04 ^aB^	8.15 ± 0.05 ^bB^	7.85 ± 0.09 ^bB^	*p* < 0.001

CON, PS without additive; LB, PS plus *L. buchneri* inoculum; KP, LB plus either of 125 g kraft pulp/kg PS; WB, 50 g wheat bran/kg PS; BP, 125 g beet pulp /kg PS. CFU, colony forming unit; FM, fresh matter; ND, not detected; and Trt, (effect of) treatment. Values are expressed as mean ± SE (n = 2 for day 0, and n = 3 for others); mean values in the same row with no common small superscripts are significantly different, assuming ND as 0; and mean values in the same column with no common large superscripts for each item are significantly different, assuming ND as 0.

**Table 3:** Fermentation characteristics of persimmon skin silage supplemented with absorbents (Experiment 1).

Items	Treatment					Contrast
	CON	LB	KP	WB	BP	
DM (g/kg FM)						
Day 0	288 ± 5 ^c^	280 ± 5 ^c^	365 ± 6 ^b^	409 ± 3 ^a^	359 ± 7 ^b^	Trt, *p* < 0.001 Time, *p* = 0.07 Trt×Time, *p* = 0.72
Day 60	281 ± 14 ^c^	261 ± 9 ^c^	323 ± 26 ^b^	388 ± 31 ^a^	355 ± 4 ^b^	
NDF (g/kg DM)						
Day 0	236 ± 4 ^c^	236 ± 4 ^c^	377 ± 11 ^a^	336 ± 3 ^b^	327 ± 6 ^b^	Trt, *p* < 0.001 Time, *p* = 0.73 Trt×Time, *p* = 0.06
Day 60	196 ± 62 ^cd^	167 ± 34 ^d^	494 ± 23 ^a^	338 ± 21 ^b^	287 ± 37 ^c^	
After 60 days ensiling						
Lactate (g/100 g DM)	3.51 ± 0.19 ^b^	3.39 ± 0.17 ^b^	2.99 ± 0.11 ^c^	4.39 ± 0.29 ^a^	4.88 ± 0.31 ^a^	*p* = < 0.001
Acetate (g/100 g DM)	3.52 ± 0.09	6.07 ± 0.27	4.15 ± 0.07	4.76 ± 1.03	5.16 ± 1.47	*p* = 0.08
NH_3_-N (mg/g Total N)	0.31 ± 0.11	0.20 ± 0.18	0.09 ± 0.07	0.06 ± 0.07	0.48 ± 0.15	*p* = 0.07
Effluent (mL/100 g FM)	21.3 ± 1.3 ^a^	17.5 ± 2.5 ^a^	0 ^b^	0 ^b^	0 ^b^	*p* < 0.001
DM loss in effluent (g/100 g DM)	12.1 ± 0.4 ^a^	12.0 ± 0.5 ^a^	0 ^b^	0 ^b^	0 ^b^	*p* < 0.001
Gas (g/100 g FM)	0.37 ± 0.02 ^a^	0.33 ± 0.03 ^ab^	0.22 ± 0.03 ^bc^	0.27 ± 0.04 ^abc^	0.17 ± 0.04 ^c^	*p* = 0.045

CON, PS without additive; LB, PS plus *L. buchneri* inoculum; KP, LB plus either of 125 g kraft pulp/kg PS; WB, 50 g wheat bran/kg PS; BP, 125 g beet pulp /kg PS. DM, dry matter; FM, fresh matter; N, nitrogen; NDF, neutral detergent fiber; and Trt, (effect of) treatment. Values are expressed as mean ± SE (n = 2 for day 0, and n = 3 for others); mean values in the same row with no common superscripts are significantly different.

**Table 4:** Effects of including ensiled or fresh persimmon skin with absorbents on the in vitro incubation characteristics (Experiment 2).

Items	BD	PSA	PSS	*p*
Total gas production (mL)	13.9 ± 1.5	16.6 ± 0.9	15.1 ± 1.3	0.118
Total VFA (mmol/L)	91.6 ± 7.5	80.5 ± 4.8	94.2 ± 8.2	0.108
Acetate (mol /100 mol total VFA)	52.3 ± 1.2 ^a^	46.7 ± 0.2 ^b^	52.4 ± 0.4 ^a^	<0.001
Propionate (mol /100 mol total VFA)	35.3 ± 2.6 ^b^	39.9 ± 1.3 ^a^	40.6 ± 0.8 ^a^	0.019
Butyrate (mol /100 mol total VFA)	12.2 ± 3.5 ^a^	13.3 ± 1.2 ^a^	6.9 ± 0.5 ^b^	0.019
NH_3_–N (mg/L)	35.8 ± 1.4 ^a^	18.6 ± 5.5 ^b^	32.3 ± 3.3 ^ab^	0.004
Methane (mmol/L)	10.3 ± 1.1 ^a^	10.1 ± 0.7 ^a^	1.7 ± 0.4 ^b^	<0.001
Total bacteria (10^9^ copies/mL)	4.92 ± 0.98 ^a^	2.67 ± 0.25 ^b^	4.64 ± 1.16 ^a^	0.037
*Fibrobacter* (% total bacteria)	1.93 ± 0.40 ^b^	2.88 ± 0.38 ^a^	0.93 ± 0.15 ^c^	<0.001
Archaea (10^8^ copies/mL)	1.53 ± 0.34	1.71 ± 0.34	0.68 ± 0.03	0.057

BD, basal diet; PSA, basal diet with unfermented PS (with absorbents) at ratio 67:33; PSS, basal diet with ensiled PS (with absorbents) at ratio 67:33; and VFA, volatile fatty acid. Values are expressed as mean ± SE; mean values in the same row with no common superscripts are significantly different.

**Table 5:** Feed intake and ingestive behavior in animal experiment using persimmon skin silage (PSS) (main test, Experiment 3).

Items	Control Period	PSS Period	*p*
DM intake (kg/day)	1.29 ± 0.10	1.40 ± 0.04	0.441
Organic matter intake (kg/day)	1.21 ± 0.09	1.31 ± 0.04	0.428
Crude protein intake (g/day)	95.9 ± 7.0	100.9 ± 3.1	0.693
NDF intake (g/day)	723.6 ± 62.9	747.3 ± 21.7	0.905
Time spent eating (min/kg ingested DM)	164 ± 5 ^a^	130 ± 8 ^b^	0.011
Time spent ruminating (min/kg ingested DM)	414 ± 11	413 ± 14	0.950
Body weight change in each period (kg)	-1.00 ± 1.05	-1.50 ± 0.62	0.717

DM, dry matter; NDF, neutral detergent fiber. Values are expressed as mean ± SE of six animals; measured data for three days in each treatment of each animal were averaged; mean values in the same row with no common superscripts are significantly different.

## Supplementary Materials

The following are available online at https://www.mdpi.com/2076-2615/10/4/612/s1, Table S1: Detail feed composition values of test feeds prior to ensiling (Experiment 1), Table S2: Chemical composition and microbial characteristics of persimmon skin silage supplemented with absorbents manufactured in a laboratory scale, Table S3: Feed compositions for the feeding trial (Pretest, Experiment 3).
