# Supplementary material for: Fermentative Quality and Animal Acceptability of Ensiled Persimmon Skin with Absorbents for Practical Use in Ruminant Feed
Source: Animals (Basel). 2020 Apr 2;10(4):612. doi: 10.3390/ani10040612 (PMC7222718; doi:10.3390/ani10040612)
Supplement: Supplementary file 1 [file animals-10-00612-s001.pdf]

Table S1. Detail feed composition values of test feeds prior to ensiling (Experiment 1).

	CON and LB	KP	WB	BP
Soluble protein (g/kg DM)	25	22	34	24
NDF (g/kg DM)	236	331	284	269
ADF (g/kg DM)	95	206	107	117
ADL (g/kg DM)	15	20	19	23
Hemicellulose (g/kg DM)	141	126	177	152
Cellulose (g/kg DM)	80	185	89	93

CON, PS without additive; LB, PS plus *L. buchneri* inoculum; KP, LB plus either of 125 g kraft pulp/kg PS; WB, 50 g wheat bran/kg PS; BP, 125 g beet pulp /kg PS. ADF, Acid detergent fiber; ADL, Acid detergent lignin; NDF, Neutral detergent fiber. Hemicellulose = NDF - ADF; Cellulose = ADF-ADL. As mentioned in Materials and Methods, values except for NDF were the calculated ones with reference to those in each material (PS, KP, WB, and BP). The amount of inoculated LAB was excluded from the calculation.

Table S2. Chemical composition and microbial characteristics of persimmon skin silage supplemented with absorbents manufactured in a laboratory scale.

	For batch culture test (Experiment 2) and Pretest (Experiment 3)	For Main test (Experiment 3)
Ensiling duration (days)	60	21
Values post ensiling		
DM (%)	34.0	31.5
pH	3.60	3.60
Yeast (log10 CFU/g FM)	Not detected	Not detected
Lactic acid bacteria (log10 CFU/g FM)	8.33	8.42
Effluent (mL/100g FM)	0	0

CFU, colony forming unit; DM, dry matter; FM, fresh matter.

Table S3. Feed compositions for the feeding trial (Pretest, Experiment 3).

Item	Treatment		
	0 % PSS	12%PSS	25% PSS
Hay cube (g/ kg DM)	586	621	656
Wheat bran (g/ kg DM)	413	191	0
Persimmon skin silage (g/ kg DM)	0	122	245
Soybean meal (g/ kg DM)	0	64	97
DM (g/kg FM)	881	756	661

PSS, persimmon skin silage. DM, dry matter; FM, fresh matter.
